# Comparison of implementation strategies to influence adherence to the clinical pathway for screening, assessment and management of anxiety and depression in adult cancer patients (ADAPT CP): study protocol of a cluster randomised controlled trial

**DOI:** 10.1186/s12885-018-4962-9

**Published:** 2018-11-07

**Authors:** Phyllis Butow, Joanne Shaw, Heather L. Shepherd, Melanie Price, Lindy Masya, Brian Kelly, Nicole M. Rankin, Afaf Girgis, Thomas F. Hack, Philip Beale, Rosalie Viney, Haryana M. Dhillon, Joseph Coll, Patrick Kelly, Melanie Lovell, Peter Grimison, Tim Shaw, Tim Luckett, Jessica Cuddy, Fiona White, The ADAPT Program Group, Phyllis Butow, Joanne Shaw, Melanie Price, Heather Shepherd, Lindy Masya, Nicole Rankin, Afaf Girgis, Brian Kelly, Thomas Hack, Rosalie Viney, Josephine Clayton, Philip Beale, Laura Kirsten, Haryana Dhillon, Peter Grimison, Michael Murphy, Jill Newby, John Stubbs, Frances Orr, Toni Lindsay, Gavin Andrews, Tim Shaw, Joseph Coll, Karen Allison, Jessica Cuddy, Fiona White, Liesbeth Geerligs, Melanie Lovell, Tim Luckett, Kate Baychek, Don Piro, Alison Pearce, Jackie Yim

**Affiliations:** 1 Psycho-Oncology Co-operative Research Group (PoCoG), University of Sydney, Sydney, NSW 2006 Australia; 2 School of Medicine and Public Health, University of Newcastle, Newcastle, NSW Australia; 3 Cancer Council NSW, Woolloomooloo, NSW Australia; 4 Centre for Oncology Education and Research Translation (CONCERT), Ingham Institute for Applied Medical Research, South Western Sydney Clinical School, University of New South Wales, Kensington, Australia; 5 College of Nursing, Rady Faculty of Health Sciences, University of Manitoba, Winnipeg, Canada; 6 CancerCare Manitoba Research Institute, Winnipeg, Canada; 7 Cancer Services for the Sydney Local Health District (Incorporating Royal Prince Alfred, Concord and Canterbury Hospitals, Campsie, NSW Australia; 8 Centre for Health Economics Research and Evaluation, University of Technology, Sydney, NSW Australia; 9 Centre for Medical Psychology and Evidence-based Decision-making (CeMPED), University of Sydney, Sydney, NSW Australia; 10 School of Public Health, University of Sydney, Sydney, NSW Australia; 11 HammondCare Northern Sydney, Sydney, NSW Australia; 12 Northern Clinical School, University of Sydney, Sydney, NSW Australia; 13 Chris O’Brien Lifehouse, Camperdown, NSW Australia; 14 Sydney Medical School, University of Sydney, Sydney, NSW Australia; 15 Charles Perkins Centre Faculty of Health Sciences, University of Sydney, Sydney, NSW Australia; 16 Improving Palliative, Aged and Chronic Care through Clinical Research and Translation (IMPACCT), University of Technology Sydney, Ultimo, NSW Australia

**Keywords:** Implementation, Clinical pathways, Cancer, Anxiety and depression, Cluster randomised controlled trial, Health services, Psycho-oncology

## Abstract

**Background:**

Health service change is difficult to achieve. One strategy to facilitate such change is the clinical pathway, a guide for clinicians containing a defined set of evidence-based interventions for a specific condition. However, optimal strategies for implementing clinical pathways are not well understood. Building on a strong evidence-base, the Psycho-Oncology Co-operative Research Group (PoCoG) in Australia developed an evidence and consensus-based clinical pathway for screening, assessing and managing cancer-related anxiety and depression (ADAPT CP) and web-based resources to support it - staff training, patient education, cognitive-behavioural therapy and a management system (ADAPT Portal). The ADAPT Portal manages patient screening and prompts staff to follow the recommendations of the ADAPT CP. This study compares the clinical and cost effectiveness of two implementation strategies (varying in resource intensiveness), designed to encourage adherence to the ADAPT CP over a 12-month period.

**Methods:**

This cluster randomised controlled trial will recruit 12 cancer service sites, stratified by size (large versus small), and randomised at site level to a standard (Core) versus supported (Enhanced) implementation strategy. After a 3-month period of site engagement, staff training and site tailoring of the ADAPT CP and Portal, each site will “Go-live”, implementing the ADAPT CP for 12 months. During the implementation phase, all eligible patients will be introduced to the ADAPT CP as routine care. Patient participants will be registered on the ADAPT Portal to complete screening for anxiety and depression. Staff will be responsible for responding to prompts to follow the ADAPT CP. The primary outcome will be adherence to the ADAPT CP. Secondary outcomes include staff attitudes to and experiences of following the ADAPT CP, using the ADAPT Portal and being exposed to ADAPT implementation strategies, collected using quantitative and qualitative methods. Data will be collected at T0 (baseline, after site engagement), T1 (6 months post Go-live) and T2 (12 months post Go-live).

**Discussion:**

This will be the first cluster randomised trial to establish optimal levels of implementation effort and associated costs to achieve successful uptake of a clinical pathway within cancer care.

**Trial registration:**

The study was registered prospectively with the ANZCTR on 22/3/2017. Trial ID ACTRN12617000411347

## Background

Anxiety and depression are the third largest causes of disability in Australia, impacting on family and social functioning, work performance, suicidal ideation, and survival [1, 2]. Rates of anxiety and depression are significantly higher in cancer patients than in the general population with point prevalence estimates of 20.7% for any mood disorder [3]. Despite high acceptance that psychosocial care is integral to quality cancer care, anxiety and depression are often undetected and their impact on patients under-estimated [4] in busy cancer services. Consequently a high unmet need for psychosocial care is persistent in cancer patients [5], despite the large evidence-base that interventions for anxiety and depression in patients with cancer are effective in the short and long-term [6, 7].

Because anxiety and depression are often under-detected, routine screening of all cancer patients for psychological distress using validated, reliable, objective measures, is internationally endorsed [8]. The International Psycho-Oncology Society (IPOS) and 68 affiliated organisations have set a standard of care that calls for monitoring distress as the “6th vital sign”. In Canada, cancer services have demonstrated that national screening of patients for distress is acceptable, feasible, and can be implemented [9], yet in Australia screening is not standard practice, and occurs to a variable extent across services.

Screening and detection, however, are only part of the solution. Several recent systematic reviews [10–12] highlighted the most significant predictor of improved patient outcomes as receipt of appropriate care after screening [10, 12]. Yet in the studies evaluated, only one in three patients received treatment after a positive screen for distress. In Australia, patterns of referral, treatment and follow-up of detected anxiety and depression vary across services. To address under-referral and inadequate management, a clear, empirically validated, clinical pathway to manage anxiety and depression is needed. Clinical pathways are standardised, evidence-based multidisciplinary management plans, which identify an appropriate sequence of clinical interventions, timeframes, milestones and expected outcomes for one or more patient groups. Clinical pathways have been shown to improve patient outcomes [13].

### ADAPT clinical pathway: background and development

A clinical pathway for screening, assessment and management of anxiety and depression in adult cancer patients (ADAPT CP) was developed to guide best practice in Australia [14]. The ADAPT CP is based on a review of the evidence and was refined through comprehensive stakeholder review and a Delphi consensus process [15]. Self-management and patient empowerment are built into the ADAPT CP and are important principles underlying its structure. The ADAPT CP follows a stepped care model incorporating iterative screening at recommended intervals, with triage to one of five steps (from universal care and self-management for those with minimal or mild levels of anxiety and/or depression, to specialist care for those with severe anxiety and/or depression), with review and change in step where necessary. Evidence-based recommendations on staff responsibilities, and content and timing of interventions, are provided for each step and tailored to available resources [14].

The ADAPT CP recommends formalised routine screening for anxiety and depression using the Edmonton Symptom Assessment System (ESAS-R) [16] or Distress Thermometer (DT) [17] as brief screening tools, combined with a more detailed tool, such as the Hospital Anxiety and Depression Scale (HADS) [18], to identify likely caseness (a clinical diagnosis). A structured clinical interview is required to confirm diagnosis.

While clinical pathways have shown considerable success in bringing about change in patient management, the context and implementation strategies utilised when introducing them into routine care are critical to success [19, 20]. Failed intervention implementation efforts can disrupt work flows, add significant staff burden and increase staff resistance to future interventions [21]. The Promoting Action on Research Implementation in Health Sciences (PARiHS) framework posits that successful implementation occurs when evidence is robust, the context receptive to change, and the change process appropriately facilitated with care taken to address barriers [22]. We conducted a barrier analysis [23] which, similar to studies outside the cancer setting [24, 25], identified lack of resources, education and training and support from leaders, poor uptake by patients, and lack of integration within the community, as key.

### ADAPT resources

In response to these identified barriers, our team developed and tested a number of online resources to support implementation of the ADAPT CP, including a web-based management system to operationalise the pathway tailored to site-specific staffing and resources (the ADAPT Portal), patient education materials, health professional education modules and an online cognitive-behavioural therapy program for anxiety and depression in patients with cancer (iCanADAPT) [26].

*The ADAPT Portal* prompts patients to complete validated screening and assessment questionnaires; alerts health professionals by system flags and email when a patient has elevated anxiety or depression; describes the pathway relevant to that individual patient and appropriate staff responses; provides standardised templates for referral and progress review to/from mental health services when required; and schedules ongoing follow up screening of patients at pivotal times/visits. The ADAPT Portal was pilot-tested in early 2017 (paper under review) and shown to be feasible, technically operational and acceptable.

*The ADAPT patient education materials* explain the importance of screening for anxiety and depression, the process involved for patients in the use of the ADAPT CP and Portal, and links to existing patient/caregiver resources about cancer-related anxiety and depression. A pilot study using qualitative methods with 20 current cancer patients and survivors showed the materials to be acceptable, and at an appropriate reading level (paper under review).

*The ADAPT online health professional education modules* were developed using evidence-based learning principles and spaced education to promote interactive learning. This education targets screening, assessing severity, making a referral - particularly when patients are reluctant - and empathic communication. Modules are available on eviQ Cancer Education Online (www.eviq.org.au), a widely used Oncology education site in Australia, via links in the ADAPT Portal. These education modules were pilot tested in a pre-post simulation study with 12 Oncology nurses and found to be acceptable, feasible and to associated with increased skills (paper in preparation).

*A program delivering online cognitive-behavioural therapy* for anxiety and depression has been developed for patients with early stage cancer (iCanADAPT Early). The therapy includes behavioural activation, cognitive restructuring and problem solving, and mindfulness strategies, completed in 6–8 sessions over 10–12 weeks. This online program is available in the ADAPT Portal for patient self-referral or clinician referral. Participants complete a short screening instrument (the K-10) [27] before each online lesson. A clinical psychologist monitors the progress of participants enrolled in iCanADAPT and contacts those with persistently elevated scores to assess need for referral to face-to-face therapy. iCanADAPT Early has been piloted [26] and evaluated in a randomised controlled trial and shown to be effective in reducing anxiety and depression compared to a care-as-usual control group (paper under review).

### Implementing the ADAPT clinical pathway in cancer services

There is a growing evidence-base that various strategies (such as presenting evidence that the intervention is effective; local, respected champions to promote the intervention; systems that reduce the burden of implementation as much as possible; and ongoing audit and feedback on implementation adherence and success) increase the likelihood of successful implementation [28]. However, there is little clarity regarding the optimal combination and dose effect of implementation strategies, and almost no data about the costs of implementation. Without such data it is difficult for health systems to incorporate CPs into routine delivery of care.

In this study we aim to determine the optimal “dose” of implementation effort and cost required to effectively achieve adherence to the ADAPT CP. Thus, cancer services participating in the study will be randomised to a standard (Core) implementation strategy versus a supported, tailored (Enhanced) implementation strategy for the ADAPT CP.

## Methods and design

### Primary objective

The primary objective of the ADAPT Cluster randomised controlled trial (Cluster RCT) is to evaluate the efficacy and cost-effectiveness of two implementation strategies (Core versus Enhanced) in achieving adherence to the ADAPT CP.

### Secondary objectives

Secondary objectives are to:

i. describe adherence to each component of the ADAPT CP and predictors of adherence
ii. describe the acceptability, adoption, appropriateness, feasibility and fidelity of individual implementation strategies and resources used to support implementation of the ADAPT CP in cancer services in NSW
iii. evaluate staff and patient acceptability of the ADAPT CP, within a NSW health context
iv. describe the costs of the ADAPT CP and compare the costs of the two implementation strategies (Core vs. Enhanced) used to facilitate uptake of the ADAPT CP
v. evaluate the cost effectiveness of two implementation strategies (Core vs. Enhanced) used to facilitate uptake of the ADAPT CP
vi. describe levels of anxiety and depression of patients participating in the ADAPT CP over a 12-month period, and patients’ allocation to five clinical, subclinical and non-clinical categories of anxiety and depression by staff
vii. describe referral patterns of cancer services before and after the introduction of the ADAPT CP, including uptake of referrals by patients.

### Hypotheses

(Note, methods used to measure each outcome referred to in the hypotheses are described in the Measures sub-section of the Methods and design section below.)

#### Primary hypothesis

Adherence to the ADAPT CP (yes/no, defined as having completed 70% or more of the recommended ADAPT CP components) will be greater in the Enhanced implementation arm than in the Core implementation arm.

#### Secondary hypotheses

- Adherence to the ADAPT CP and predictors of adherence
  1. Adherence to each component of the ADAPT CP (yes/no) will be greater in the Enhanced implementation arm than in the Core implementation arm.
  2. Adherence to the ADAPT CP will increase from the 6- to 12-month follow-up.
  3. Staff-perceived individual and site readiness to change will be associated with adherence to the ADAPT CP.
  4. Staff use of the ADAPT resources will be associated with adherence to the ADAPT CP.
  5. Characteristics of the service (size, psychosocial staffing, existing psychosocial screening), will influence staff attitudes and adherence to the ADAPT CP.
  6. Greater awareness/use and perceived utility of implementation strategies will be associated with better adherence to the ADAPT CP.
- Acceptability of the ADAPT CP and resources
  7. The ADAPT CP will be acceptable to staff and patients.
  8. At least 60% of staff will be aware of, use, and rate as useful, the ADAPT resources (including the health professional training and the ADAPT Portal).
- ADAPT implementation strategies (Core and Enhanced)
  9. Both Core and Enhanced ADAPT implementation strategies will be feasible (delivered) and delivered with fidelity as planned.
  10. At least 60% of staff will be aware of, use, and rate as useful, both Core and Enhanced ADAPT implementation strategies.
  11. Acceptability, adoption, appropriateness, feasibility, fidelity, and penetration of implementation strategies will be greater in the Enhanced implementation arm than the Core implementation arm.
  12. Improvements in staff-reported acceptability of the ADAPT CP and perceived readiness to change will be greater at 6- and 12-month follow-ups in the Enhanced implementation arm compared to the Core implementation arm.
- Cost-effectiveness
  13. While more costly, the Enhanced implementation arm will achieve greater adherence than the Core implementation arm.
- Patient outcomes
  14. Anxiety and depression screening scores at rescreening will be significantly lower than at the initial screen.

### Study design and setting

The study is a Cluster RCT being undertaken in the state of New South Wales (NSW), Australia. NSW health services are managed through 15 local health districts and three specialty networks. Twelve cancer services will be stratified by size of service - large (≥100 new pts./year) versus small (< 100 new pts./year)) to ensure equal patient volume in the two study arms. The sites will then be randomised at the cancer service level to a Core implementation strategy arm versus an Enhanced implementation strategy arm to facilitate uptake of the ADAPT CP.

After a 3-month period of site engagement, staff training and tailoring of the ADAPT CP and ADAPT Portal for each participating cancer service (site), that site will “go-live” and implement the ADAPT CP for 12 months, as part of routine care. ADAPT research staff will deliver the Enhanced or Core implementation strategies over that period, according to randomisation. The study uses a mixed methods design. Both quantitative and qualitative data eliciting staff attitudes to and experiences of the ADAPT CP and implementation strategies utilised, will be collected at T0 (baseline - after site engagement, at ADAPT go-live), T1 (6 months post go-live) and T2 (12 months post go-live).

Taking into account the governance, engagement and implementation phases, each site will participate in the study for a period of 18 months. Three sites will be activated every 3 months. Thus, all sites will have completed the study over a 30 month/2.5-year period.

#### Inclusion criteria

Cancer services will be eligible to participate if they provide care for at least 100 patients per year in NSW Australia. Eligible cancer services may operate within the public or private healthcare system. They may be whole services or single departments within services, such as tumour streams (e.g. breast cancer or haematology) or treatment streams (such as medical or radiation oncology). If there is more than one department/hospital which shares common staff members, they will be considered one site for the purpose of this study. This is necessary to ensure that participating staff members are not exposed to both Core and Enhanced implementation strategies.

#### Exclusion criteria

Cancer services will be excluded if they are unable to commit to:

- endorsing and enabling staff training for ADAPT
- engaging with ADAPT staff for the 3-month engagement and tailoring period
- allowing a complete site profile audit and release of their organisational chart
- authorising release (with individual patient consent) of Medical and Pharmaceutical Benefit (MBS/PBS) provider information and statistics;

and if they do not have Wi-Fi/broadband/internet access and/or an internet browser version that complies with the ADAPT Portal requirements.

#### Recruitment: cancer services and staff

An initial meeting (face to face or via teleconference) will be held, attended by representatives of the ADAPT research team, the Head of the cancer service, and representatives of the psychosocial, nursing, clinical trial and IT staff. At this meeting, the study will be described in detail, and potential interest in participation will be ascertained. Interested cancer services will be asked to identify a local ADAPT champion, and to sign an ADAPT Research Participation Agreement.

All staff employed on a permanent or ongoing (6 months or more) basis, either full or part time or as a visiting medical officer, in a role that provides clinical care to patients with cancer or client focussed administrative support or IT/managerial support, will be invited to participate in the study. Staff will be identified as part of the organisation chart mapping exercise during site engagement. Site champions will identify and approach eligible staff, to whom they will explain the study. Interested staff will be contacted by the research team and provided with a written information sheet and consent form to sign.

A site, or individual cancer service staff member, may withdraw from the study and elect to have their data removed from the study database. If a site chooses to withdraw prior to completion of baseline data collection, and there is sufficient time within the study timeline, the site will be replaced by a reserve list site pre-randomised to the same study arm.

#### Recruitment: patients

All patients at participating sites receiving psychosocial care related to their cancer diagnosis will be treated according to the ADAPT CP as part of routine care during the 12-month implementation period. If a patient does not wish to complete screening on a particular occasion (defer) or declines screening completely, this will be recorded in the ADAPT Portal and noted within the patient’s medical record, in line with usual record keeping practices at participating cancer services.

Patients participating in the ADAPT CP will be asked to give informed consent for the researchers to access their medical records and/or Medicare and Pharmaceutical Benefits Scheme (MBS/PBS) claims history for a period of 10 years to enable health economic analyses. Patients may withdraw this consent at any time.

### Interventions/groups

Sites will be randomised to a Core implementation arm versus an Enhanced implementation arm to facilitate uptake of the ADAPT CP. All cancer services will receive access to, and training to support use of, the online resources developed to support ADAPT. These resources (described in detail above) are the:

- ADAPT Portal
- ADAPT Health professional training
- ADAPT Patient education materials
- iCanADAPT Cognitive-behavioural Therapy

The Core implementation and Enhanced implementation arms will differ in the implementation strategies used to support the use of ADAPT CP at their cancer service. Specifically, these differences are:

- *Core:* These sites will receive implementation strategies consistent with usual roll out of a clinical pathway in the NSW health context, to inform staff of the ADAPT CP and enable them to initiate use of the ADAPT Portal and intervention resources. Once the ADAPT Portal is launched and the implementation phase has commenced, Core sites will receive reporting feedback and audit reports at 4 weekly intervals and additional support from the ADAPT team only at their request (a passive approach).
- *Enhanced:* These sites will receive the same implementation strategies as the core group, with the addition of more prolonged, active engagement of the ADAPT team, that will continue during the 12-month implementation phase. These additional strategies will include monthly meetings to identify training and support needs of local champions, additional awareness activities, audit and feedback reports prior to and midway through the implementation phase summarised and presented face-to-face by research staff to site staff, and other tailored strategies to address the specific barriers and facilitators in each site (an active approach).

The strategies, described in detail in Table 1, were developed by the research team, comprising experts in the fields of psycho-oncology and implementation science, cancer services leaders, and consumers. A literature review, NSW Health current implementation practices, and the ADAPT barriers and enablers study [23] guided the development of the strategies.

**Table 1:** Implementation strategies

Strategy	Both Core and Enhanced implementation strategies arms	Enhanced implementation strategies arm *only*
Awareness campaign	• Roadshow presentation by ADAPT staff at the site 8 weeks before “go-live” • Poster displayed prominently 4 weeks prior to and at “go-live,” (T0) • Email from site champion to all staff 1 week before “go-live” (T0)	• Additional posters at 4-monthly intervals during implementation • Newsletters emailed to site staff at 2, 4, 6, 9 and 12 months
Champions	• Champion identification and role definition • Provision of email templates for champion to send staff • Inclusion of champion contact details in all staff communication	• Additional proactive contact with Champions at monthly intervals to discuss progress, provide audit reports and discuss additional implementation strategies as needed
Academic detailing and support	• Sites provide with a written report summarising change readiness data from baseline staff interviews at T0 • Tailoring of the ADAPT Portal to site requirements during engagement meetings • Study close meeting with all key staff to discuss sustainability of the ADAPT CP	• Sites provided with a verbal report of these data, at T0, T1 and T2 • Quarterly review of ADAPT Portal configuration to confirm allocated responsibilities and service tailoring
Reporting	• Monthly written reports on Portal statistics	• Presentation and highlighting of issues from monthly reports in face-to-face meetings with site
Technological support	• IT support for the ADAPT Portal	

### Study procedures and timeline

Randomisation will be performed by the study statistician using online randomisation software (STATA). Allocation concealment will be preserved; both sites and ADAPT Program staff conducting assessments will remain blinded until study completion. Following randomisation, sites will undertake a 3-month period of engagement to clarify study procedures, train staff in the ADAPT CP and use of the ADAPT resources, and tailor the ADAPT Portal to local site resources and preferences. Study sites will then complete baseline (T0) staff questionnaires and interviews to ascertain staff views on service and staff readiness to adopt the ADAPT CP. The online questionnaires will be available on RedCap (Research Electronic Data Capture) [29] and automatically downloaded to a database to reduce possible bias. The ADAPT research team will follow-up those participants who do not complete or return their questionnaire within 2 weeks, with up to three reminder emails or phone calls at different times of the day. A subset of staff with key roles in implementing the ADAPT CP will be invited to participate in an audio-recorded semi-structured interview exploring their views of the ADAPT CP, barriers and facilitators to its use and site readiness.

Sites will then “go-live,” integrating the ADAPT CP into clinical care for a 12-month implementation period. Staff will be encouraged to introduce the ADAPT CP to all patients as part of routine care, and on their verbal consent, register them into the ADAPT Portal. Patients will then be asked to access the ADAPT Portal for efficient screening and data collection, information and access to ADAPT resources. Staff will be asked to respond to ADAPT emails and alerts for patients who require further assessment and referral. ADAPT research staff will administer Core versus Enhanced implementation strategies as per randomisation during this period.

Mid-way through the 12-month implementation period (T1 at 6 months) and at the end of the implementation period (T2 at 12 months), questionnaire data will again be collected from cancer service staff, and a subset of cancer service staff will participate in an audio-recorded semi-structured interview to ascertain staff views on implementation outcomes, and awareness of and perceived utility of the ADAPT CP implementation strategies.

Timing of enrolment, engagement, intervention and data collection is shown in Table 2.

**Table 2:** Schedule of enrolment, engagement, intervention and data collection

		Study period					
	Enrolment	Engagement	Allocation	Intervention			Costs retrieval
Timepoint	-T2	-T1	0	T0 “Go-live”	T1 6 mths	T2 12 mths	T3 3 mths post
Enrolment:							
Eligibility Screen	X						
Informed Consent	X						
Governance	X						
Engagement:							
Role definition		X					
Tailoring		X					
Staff training		X					
Allocation			X				
Interventions:							
Core				X	X	X	
Enhanced				X	X	X	
Assessments:							
Primary outcome							
Adherence: CP					X	X	
Secondary outcomes							
Acceptability				X	X	X	
Adoption					X	X	
Appropriateness				X	X	X	
Feasibility				X	X	X	
Costs				X	X	X	X
Penetration					X	X	
Sustainability						X	
ORIC, Hands4U, study-developed organisational readiness items				X	X	X	
Demographic & disease items				X			

Case report forms (CRFs) in this study will take the form of site profile forms, contact logs, implementation strategy checklist and observational diaries, implementation strategy data psycho-social activity data and Medicare Data forms completed by ADAPT Program staff to collect service level data and staff surveys completed by cancer service staff participants. All other quantitative data will be collected electronically via the ADAPT portal. Additionally, the research team will conduct internal audits by reviewing automated reports built into the ADAPT Portal to ensure system functioning, correctness and accuracy of data entry.

### Measures

#### Primary outcome

The primary outcome is site adherence to the ADAPT CP, operationalised as the percentage of patients at each site whose care is considered adherent to the ADAPT CP, using data extracted from the ADAPT Portal. The measure, guided by the literature on adherence [30], captures for each patient, whether they received the ADAPT CP components (screening, triage conversation, referral, referral uptake check, progress review, discharge summary, and rescreening) appropriate for their level of anxiety and depression, expressed as a continuous score of 0–100. Mean scores will be calculated for each site, averaging scores for all patients. This continuous mean score will be transformed into a binary score of adherent (≥70% of patients experiencing ≥70% of appropriate components) or non-adherent (< 70% of patients experiencing ≥70% of appropriate components), based on accepted implementation targets [31, 32].

#### Secondary outcomes

ADAPT CP secondary outcomes will address the following domains: acceptability, adoption, appropriateness, feasibility, fidelity, cost and penetration, and sustainability of the ADAPT CP, defined in large part by the Proctor framework of implementation outcomes [33] (see detail below and in Table 1). ADAPT implementation strategy outcomes will address similar domains, but with reference to the degree to which implementation strategies (Awareness Campaigns, Champions, Academic Detailing and Support, Education, Reporting and Technology) support the implementation of the ADAPT CP.

*Acceptability is* operationalised as the perception of cancer service staff that the ADAPT CP (1) and the ADAPT implementation strategies (2) are agreeable, palatable and satisfactory, the first in addressing anxiety and depression in their client population, the second in facilitating implementation. This will be measured qualitatively during staff interviews.

*Adoption* of the ADAPT CP (1) is operationalised as the proportion of patients at each site who are registered and screened on the ADAPT Portal. Adoption of the ADAPT implementation strategies (2), is operationalised as mean staff ratings of how much they were aware of, observed and used each implementation strategy, collected via the staff questionnaire.

*Appropriateness* is operationalised as the extent to which staff believe that the ADAPT CP has fit, relevance and compatibility at the level of their setting, their role, and the needs of their consumers, measured quantitatively by the Hands 4 U questionnaire [34] (items adapted to the context of this study) and additional study-developed items within the staff questionnaire. Appropriateness will also be measured qualitatively in staff interviews.

*Feasibility*, operationalised as the extent to which the ADAPT CP is considered feasible by staff of each service, given their different needs and resources, and the degree of individual tailoring required to set up the ADAPT Portal, will be measured qualitatively in semi-structured interviews.

*Direct costs* of delivering the ADAPT implementation strategies (in terms of research staff time and resources) will be calculated from the research team logs. Costs of developing, maintaining and delivering different components of the ADAPT CP, will also be calculated. Patient health services use, including pharmaceutical prescriptions related to anxiety and depression, will be extracted from Medicare MBS/PBS and hospital medical records data. These data will be used to estimate changes in health system resource use before and after participating in the ADAPT CP, and to determine whether differences in adherence are associated with differences in health system resource use and overall costs.

*Penetration* is operationalised as the number of staff and services who were aware of, and engaged with, the ADAPT CP and implementation strategies.

*Sustainability*, defined as the extent to which the ADAPT CP is maintained or institutionalized within a service setting’s ongoing, stable operations, will not be measured during the ADAPT Cluster RCT, as no data will be collected beyond the 12 months implementation phase. However, intention to sustain the ADAPT CP will be elicited during T2 qualitative interviews. Further funding will be sought to audit actual sustainability in the future at participating sites, post the RCT.

**Predictors of adherence**, including staff attitudes to the ADAPT CP, perceived social influence to enact it, self-efficacy, organisational readiness and intention to implement the ADAPT CP, will be assessed by the Organisational Readiness to Change (ORIC) questionnaire [35], Hands4U questionnaire [34] and some study-developed ADAPT organisational readiness items.

### Potential confounders

***Demographic and disease factors*** will be collected including years in oncology practice and years in current position (staff only); age, gender, marital status, education, postcode, ATSI status (staff and patients); and cancer type and stage of disease (patients only).

**Additional secondary outcomes** include the prevalence of anxiety and depression over time, accessed from the ADAPT Portal.

### Safety reporting

All adverse events (AEs) and serious adverse events (SAEs) related to implementing the ADAPT CP will be recorded concisely using standard terms.

The interventions being evaluated in this study are targeted at cancer service staff. It is not envisaged that cancer service staff will experience any adverse events. In the unlikely occurrence that attendance at a training or information session, completion of a survey or an interview does result in an adverse event, the relevant ethics committee will be notified. As screening and management of anxiety and depression will be introduced as standard practice, the reporting of adverse events related to anxiety and depression will not be reported as a study related AE event. There are no safety endpoints and no criteria for early study termination.

### Data management

All data will be stored in locked filing cabinets in a locked office at The University of Sydney or scanned and stored in password protected files and kept at the University of Sydney on a secure server. Electronic data will be password protected and kept at The University of Sydney on a secure server, which is backed up daily. Study-related records for all participants will be retained in a secure storage facility for at least 7 years after the completion of the research, according to the National Health and Medical Research Council requirements. These include: participant files, study protocol, signed consent forms, CRFs, questionnaires, ethics correspondence and approvals, other regulatory documentation, and other documents pertaining to the conduct of the study. All relevant documentation according to the Data Management Plan will be held by the lead investigator at each participating site for the duration of the study. This study will adhere to PoCoG’s quality assurance processes, including adherence to standard operating procedures and regularly reporting of study progress to the organisation.

### Statistical methods

#### Sample size and justification

##### Power for primary endpoints

Based on cancer registry data for NSW (2009) (https://www.cancerinstitute.org.au/data-research/data-held-by-cinsw/nsw-cancer-registry), approximately 37,525 patients in NSW will be diagnosed with cancer in NSW each year. Based on prior epidemiological studies, 42–45% and 35–37% of these will experience mild depression or mild anxiety, respectively [3, 36, 37] and 22–36% and 20–30% will experience moderate to severe depression and/or anxiety respectively.

Based on incidence and prevalence of cancer in NSW, a conservative estimate of 464 screening events per site (total sample of 5568 screening events in 12 sites), there is at least 80% power to detect a difference in adherence rates of 30% in the Core and 60% in the Enhanced arm using a two-tailed test with Type 1 error rate of 5%. The sample size has been adjusted for the variance inflation factor due to the clustered design and can accommodate an intraclass correlation coefficient (ICC) as high as 0.14 to achieve 80% power and 0.10 to achieve 90% power [38, 39]. Although estimates of the ICC are not available, we expect the ICC will be within this range. Based on the Cancer Institute figures, we do not envisage any difficulty in recruiting the required sample for this study. Note that the number of screening events and projected sample size and power may be revised based on screening at the first three services.

##### Power for secondary endpoints

Secondary outcomes will use data from either the ADAPT Portal, relating to patient activity through the ADAPT CP, MBS/PBS, staff surveys, staff interviews, or ADAPT research team observational records. Given the expected large sample size of 5568 patients, power should be adequate to test the patient activity related secondary outcomes. For secondary outcomes using staff reported data we anticipate a minimum of 10 staff per site, with 12 sites participating (120) in total.

### Primary analyses

The primary analysis will be conducted using a generalized estimating equations (GEE) approach to account for the correlation of outcomes within a cluster (site) using an exchangeable covariance structure and a bias correction factor to account for the small number of clusters [39]. The dependent variable will be clinical pathway adherence and the exposure variable will be the intervention status (core versus enhanced). The unit of analysis will be at the patient level to accommodate the weighting required by unequal cluster (site) sizes. Other independent variables will be added to the model if they are independently associated with adherence and/or their inclusion in the model changes the linear coefficient of the intervention effect by more than 20% in absolute value (i.e. effect modifiers).

### Secondary analyses

Secondary outcomes for this study include the collection of qualitative and quantitative data. Qualitative data will be analysed using content and thematic analysis. This will apply to data collected from (i) cancer service staff interviews and (ii) observational diary and contact log data captured by the ADAPT research team. Qualitative data will be coded by researchers trained in qualitative methods. Initial themes and subthemes will be identified and discussed by members of the research team to establish a coding tree. Transcripts and/or diaries and/or contact logs will then be coded by a minimum of two coders. Coding discrepancies will be resolved by discussion.

Quantitative data will be analysed using descriptive statistics, univariate and multivariate analyses. Analyses will be conducted at patient level and site level. Generalized estimating equations (GEE) will also be used.

### Compliance with regulatory guidelines

This study will be conducted in compliance with the:

- International Conference on Harmonisation of Technical Requirements for Registration of Pharmaceuticals for Human Use Good Clinical Practice (ICH-GCP),
- The National Health and Medical Research Council National Statement on Ethical Conduct in Human Research,
- The Australian Code for the Responsible Conduct of Research,
- Therapeutic Goods Administration’s Australian Clinical Trial Handbook, and
- Declaration of Helsinki.

## Discussion

This study will advance the field by identifying the level of implementation support, and resource use (Core versus Enhanced) required to effectively achieve adherence to a clinical pathway for anxiety and depression in cancer patients (ADAPT CP). It will be the first study internationally to explore this research question through a rigorous cluster randomised controlled trial, where sites are randomised to different implementation strategies. The costs and consequences of the implementation strategies will also be evaluated. The study will describe predictors of adherence, including staff and service readiness for change, staff attitudes, self-efficacy, social influence and intention to implement the CP, and staff, patient and service characteristics. A strength of the study is that adherence data, as well as potential predictors and cost data, will be collected longitudinally, allowing these factors to be explored over time.

The ADAPT CP is relevant to all cancer types and cancers of any stage. The ADAPT Cluster RCT trial will recruit cancer services providing care to a range of tumour streams, and it is intended that the ADAPT CP will be applied to all patients within these services, including those treated with curative intent and those treated palliatively. Furthermore, randomisation will be stratified by size of service to ensure inclusion of both large and small cancer services, and those within both metropolitan and rural/regional areas, to determine the implementation strategies required across a range of services likely to vary in their resources and culture. While this study is restricted to the ADAPT CP, the findings will be generalisable to other CPs, providing a broader impact on health service change.

A qualitative component is included in this study to capture the complexity of the barriers and facilitators influencing uptake of the ADAPT CP, and response to the ADAPT implementation strategies. These are expected to vary across sites with different clinical and research cultures, resources, patient populations and staffing profiles, and qualitative research has value in understanding these issues.

The two levels of implementation effort tested here (Core implementation strategy versus Enhanced implementation strategy) include multiple types of implementation strategies, and it will not be possible to determine whether one versus another has had the greatest impact. However, we will be able to investigate potential mediators and moderators of intervention success, to guide hypotheses for investigation in future studies.

With an increased focus on implementation of evidence that has been costly to accrue, and the potential to improve patient outcomes immediately, clinical pathways are likely to form the basis for care in a growing number of health systems. Clinical Pathways have great potential for enhancing the translation of evidence into practice, however health system policy and planning require accurate data on the costs and size of implementation effort required to ensure their success. Armed with such data, health service administrators can better plan introduction of clinical pathways within current budget constraints. Thus, this study will provide information critical to improving healthcare outcomes.

## Abbreviations

ADAPT Clinical Pathway or ADAPT CP: Clinical Pathway for the Screening, Assessment and Management of Anxiety and Depression in Adult Cancer Patients
ADAPT Cluster RCT: A cluster randomised controlled trial comparing the effectiveness and cost effectiveness of two implementation strategies designed to encourage adherence to the ADAPT Clinical Pathway
ADAPT Portal: Web-based system to operationalise the Clinical Pathway for the Screening, Assessment and Management of Anxiety and Depression in Adult Cancer Patients
ADAPT Program: The Anxiety and Depression Pathway Program. The Translational Program Grant: A Sustainable and supported clinical pathway for managing anxiety and depression in cancer patients
